# Editorial: A 150 years' celebration of Darwin's book on human evolution and sexual selection: its legacy and future prospects

**DOI:** 10.3389/fpsyg.2023.1217889

**Published:** 2023-06-06

**Authors:** Marco Antonio Correa Varella, Catherine Salmon, Barnaby James Wyld Dixson, Marina Butovskaya, Anabela Pinto, Boguslaw Pawlowski, Carol Cronin Weisfeld, Jaroslava Varella Valentova

**Affiliations:** ^1^Department of Experimental Psychology, Institute of Psychology, University of São Paulo, São Paulo, Brazil; ^2^Department of Psychology, Director Human-Animal Studies, University of Redlands, Redlands, CA, United States; ^3^School of Psychology, University of Queensland, Brisbane, QLD, Australia; ^4^Department of Cross-Cultural Psychology and Human Ethology, Institute of Ethnology and Anthropology (RAS), Moscow, Russia; ^5^Wolfson College, University of Cambridge, Cambridge, United Kingdom; ^6^Department of Human Biology, University of Wrocław, Wrocław, Poland; ^7^Department of Psychology, University of Detroit Mercy, Detroit, MI, United States

**Keywords:** sexual selection, creativity, arts, ovulatory cycle, development, parenting, sexual dimorphism, mate preferences

Charles Darwin is considered a great naturalist and geologist, but also a great evolutionary psychologist because of his pioneering contributions concerning the evolution of the human mind and behavior. Despite persisting misunderstandings (Varella et al., 2013), the evolutionary perspective applied to understanding human mind, behavior, and culture has greatly expanded and improved since then (Sear et al., 2007; Griffiths, 2008; Tooby, 2020). This Research Topic launched in 2021 aimed at celebrating the 150 years of Darwin's *The Descent of Man, and Selection in Relation to Sex* book from 1871, in which he offered a promising and comparative research program about human evolution and its relation to sexual selection, that has led to a variety of studies, discoveries, and scientific progress. Recently, there have been many similar initiatives for the sesquicentenary celebration, including reviews (Brooks, 2021; Fritzsche et al., 2021; Richerson et al., 2021; Harman, 2022; Rosenthal and Ryan, 2022), opinion pieces (Fuentes, 2021; Whiten et al., 2021), books (DeSilva, 2021; Bertranpetit and Peretó, 2022), and Research Topic (Gavrilets et al., 2021). The majority of celebratory texts relate to human evolution in general, and only a few touch on human psychology or even sexual selection. This Research Topic fills this gap by offering a lengthy and deep exploration of sexual selection topics related to human evolution.

The collection of 20 articles accepted and published in this celebratory Research Topic includes theoretical and empirical studies that together present an updated, diverse, and comprehensive view about the ways in which sexual selection can influence the evolution of human mind and behavior. This Research Topic offers five groups of articles: (1) Theoretical contributions related to the inner workings of sexual and social selection; (2) Contributions focused on the evolution of human capacities for arts, creativity, music, and play; (3) Contributions concerned with parenting and alloparenting; (4) Contributions approaching sexual dimorphism; and (5) Contributions investigating signaling, mate preferences, and sexual strategies.

## Theoretical contributions about sexual and social selection

This collection of articles begins with a review article by Petrie. The author provides a highly accessible review article summarizing how sexual selection has shaped the evolution of sexual dimorphism in ornamental characteristics. Petrie draws on her own iconic research demonstrating sexual selection on the peacock's train as an indirect signal of genetic benefits to discuss future directions in this field of research (Petrie, 1994). While statistical models detailing how direct and indirect mechanisms operate on sexually selected traits in non-human animals were developed decades ago (Kirkpatrick and Barton, 1997; Kokko et al., 2006), they have been slow to gain traction among researchers studying humans. Petrie presents a narrative and mathematical model demonstrating sexual selection is profoundly different from natural selection, which will help researchers studying sexual selection in humans in their future research in this growing field.

Davis and Arnocky focus their attention on a well-studied topic, mate choice and the evolution of esthetics. They emphasize that the mainstream approach to studying mate choice and attractiveness adopts the Wallacean perspective of attractive traits being possible signals of individual biological quality, while the original Darwinian approach has been mostly overlooked. In Darwin's view, female mate choice was driven by feelings based mostly on esthetics *per se*, without indicating individuals' underlying qualities (direct or indirect benefits), or even being a disadvantage to the individual, as stressed by Fisher, Zahavi, and lately by Prum, whose null hypothesis on trait-preference is described in this article. They suggest that evolutionary psychologists should expand their theoretical assumptions to consider the Lande-Kirpatrick's improvements on Darwin's original model as the null hypothesis regarding intersexual selection.

Crespi et al. review the central Darwinian mechanism of mating competition among conspecifics (sexual selection) proposed as an important evolutionary force explaining the apparent enigma of exaggerated colorful or otherwise costly traits. Darwin's concept of sexual selection was further advanced by several scholars including “runaway” selection, which could happen if choice was based on comparisons favoring relative extremes. The authors then present Alexander (1990) concept of runaway social selection arguing that an arms-race competition among individuals for social skills (and weapons) was at the core of human evolution. Runaway social selection suggests that humans generated and became their own primary selective pressures in arenas of social within-group and between-group competition, allies or partner selection, mate choice, caregiver-offspring relationships, and cultural traits and social-cultural learning. Crespi et al. argue that each of these arenas of social selection drove the evolution of different, interacting dimensions of human sociality and culture, and that they merged to shape the humans inhabiting the current world.

## Papers on arts, creativity, music, and play

When Darwin argued in favor of the universality of the human mind, he highlighted the shared pleasures that populations around the world take in singing, dancing, acting, painting, tattooing, and self-decorating. Varella explored the evolved structure of artistic motivation using a large three decades-long real-life public database from university applications in Brazil. He predicted that an evolved artistic motivation would be specific, intrinsically sourced, and temporally stable. After analyzing reasons for career-choice in two studies, he found that applicants for artistic careers specifically and consistently through time reported more intrinsic reasons, such as personal aptitude, compared to applicants for non-artistic careers. He was able to provide evidence against Steven Pinker's non-adaptationist hypothesis that artistic activities are motivated by a generic “hunger for status”, given that social prestige and other extrinsic reasons were quite low in applicants for artistic careers. He also found that probably there is a singular psychological mechanism for artistic motivation intrinsically influencing all different artistic modalities.

Darwin also argued that inclinations toward music, arts and creative expressions evolved in both sexes through sexual selection as a form of courtship display. Marin and Rathgeber present data on the sexual selection hypothesis for the evolution of human musicality in an instrumental music priming study in which they tested whether musical priming increased sexual attraction as assessed through ratings of facial attractiveness and dating desirability. While an increase in dating desirability was reported by both sexes, only females reported greater ratings of attractiveness on the part of males post prime. This highlights several points. It supports a link between music/creativity and mate choice but it also echoes the findings of others that multiple cues are relevant in mate choice and some cues may be more influential in female vs. male choice as seen in the stronger effects of the music prime on females.

Varella et al. expanded on the theme of sexual selection influencing the evolution of artistic and athletic propensities by performing the first direct comparison between the strength of intersexual and intrasexual selections acting on inclinations toward literary, visual, musical, and circus arts and sports. They analyzed self-perceived talent and expertise, proxies of intersexual selection such as mate value and sociosexuality, and proxies of intrasexual selection such as aggressiveness and intrasexual competitiveness in men and women from Brazil and the Czech Republic. They found that intersexual selection was related to literary, musical, visual and circus arts in women, and to literary, circus arts, and sports in men, while intrasexual selection was related to literary, musical, visual arts, and sports in women, and to literary, musical arts, and sports in men. Sexual selection was most strongly related to artistic inclination in women, and sports in men. They conclude that cross-culturally both sexes use some artistic inclinations as ornaments and armaments, although more as ornaments in women, and armaments in men; while sports function as ornaments and armaments only in men, and as armaments in women.

General creativity as a sexually selected psychological trait in women was investigated by Galasinska and Szymkow. They used a within-subject design and indirect hormonal measures to test whether creativity (i.e., fluency, flexibility, and originality of unusual uses for everyday objects) is higher during the fertile phase of the menstrual cycle. They found that Polish women had more original ideas during the ovulatory phase, despite having roughly the same number of ideas (i.e., fluency) during other phases of the menstrual cycle. When the fertile phase was compared to all non-fertile phases combined, they also found high flexibility (i.e., breaking a pattern) of ideas during the ovulatory phase. Given that their results were not mediated by arousal or positive mood, they conclude that creativity may be a mental ornament in women, which suggests possible ancestral influences of male choice on female creativity.

Novaes and Natividade narrowed it down to ornamental creativity, the psychological capacities that produce and appreciate aesthetically conspicuous manifestations. They offered an updated and comprehensive review of the converging evidence pointing to the influence of sexual selection on creative ornamentation. They pointed out that although associations between tendencies toward ornamental creativity and individual attractiveness or mating success are good indications of the power of sexual selection, it is not sufficient for establishing with high certainty the role of sexual selection. Thus, Novaes and Natividade compiled evidence stemming from proximate mechanisms such as neurobiology, heritability and hormonal influences, ontogenetic and social factors, and phylogenetic and comparative findings from other species. By considering Tinbergen's four questions and creating a nomological network of evidence they were able to empirically fundament the conclusion that sexual selection indeed influenced the evolution of human ornamental creativity in both sexes. They have presented the gaps in the knowledge and offered routes for future research.

Moraes et al. approached playfulness, play and play-like activities through sexual selection. They noticed that while sexual selection has been applied to many activities that are considered “play” (e.g., play music, role-playing, play sports), there are few studies applying sexual selection to adult play and gaming tendencies *per se*. They conceptually differentiated between the behaviors of play and the underlying psychological capacities, such as playfulness or competitiveness. They defined, differentiated, and dissected the constituent parts of the concepts of playfulness, play, and games. The result was a compilation of converging evidence supporting the notion that both play and games are evolved capacities in human nature, such as universality, precocity, and heritability, and emphasized indications of sexual selection, such as sex differences and relationship to mating success. They concluded by offering a number of suggestions, including considering the underlying psychological components of behavior, cross-cultural and inter-specific evidence, ecology validity, and the alignment of Tinbergen's questions with cultural explanations.

## Papers on parenting and alloparenting

An unusual feature of human evolution is the reduction of overall body hair compared to other primates. The majority of theoretical and empirical research discusses how reduced body hair evolved for thermoregulation (Wheeler, 1992), reduced parasite loads (Rantala, 2007; McIntosh et al., 2017), and mate choice (Dixson et al., 2019). Amaral approaches how humans evolved reduced hirsutism from the novel perspective of parenting. Building on her previous research on this topic (Amaral, 2008), phylogenetic comparative analyses are employed testing the tensile properties of primate hair suggesting that infants clinging to body hair were crucial for their safety during maternal tripedal movement. Amaral emphasizes that safe infant caring and sexual selection might have influenced the evolution of bipedalism in humans. She then proposes that reduced hirsutism co-evolved with bipedalism in humans via natural selection in concert with other selective pressures (e.g., thermoregulation) and cultural solutions such as huddling developed for thermoregulation in the absence of pronounced body hair. Amaral invited researchers to approach the evolution of human bipedalism aligned with reduced hirsutism as a solution to a female problem of safe carrying infants, not so much a male acquisition for throwing stones, for instance.

Daly and Perry's article focused on what sexual selection theory brings to our understanding of stepparenting and stepfamily dynamics. Highlighting the problem first with reference to non-human animals, the decision for a new mate with regard to existing offspring can be summarized by the question “Kill, ignore, or adopt?” Research has suggested that the adopt option can be understood as a part of courtship in many species and this can be seen as the product of species typical ecologies. Despite this, within species variation exists, perhaps nowhere so much as in humans. Sexual selection and parental investment theory suggest testable hypotheses about what factors predict this variability in stepparental behavior. They review the fitness consequences not only in birds but in humans, noting the recurrent presence of step-parenting over historical time, viewing stepparenting as a special case of discriminative parental solicitude in response to ecological conditions that range from local sex ratios, mortality, resource availability, and individual child traits. They suggest that our understanding of stepfamily dynamics will benefit from hypothesis testing shaped by an understanding of sexual selection and discriminative parental solicitude.

The study by Semenova et al. contributes to the understanding of evolutionary dynamics in parental conflicts by exploring some under-researched cases. The study's conclusions highlight the importance of considering sex-biased asymmetry in potential fitness gains in understanding human reproductive behavior. Specifically, models that provide new insights into the evolution of parental care are presented. One interesting finding from the study is the possibility of population division as an evolutionary outcome of human mating interaction, where “reproductive defection” serves as the best response to any action by a potential sexual partner. The study's models are particularly pertinent to sexual conflict in humans, where child rearing is a long and costly process and partner defection can greatly disrupt parental investment. Semenova et al. suggest that under a certain payoff matrix, avoiding risky reproduction represents a relatively cost-effective decision for humans of both sexes. The final model predicts that individuals who adopt cooperative reproductive strategies could cluster together in geographically, linguistically, or religiously structured populations, potentially leading to population clustering. The propensity for population separation, and the biological prerequisites for the emergence of morality have not yet been considered in the literature under a parental game theoretical approach. Authors hope to spark discussions among scientists from diverse fields, leading to renewed efforts to explore the evolution of parental care in diverse environments and cultures. Overall, the study offers new perspectives and avenues for future research in the field of evolutionary biology dealing with sex differences.

## Papers on sexual dimorphism

The evolution of marked variation in olfactory cues, cutaneous characteristics, and somatotypes have only recently received attention among researchers studying sexual selection in humans (Štěrbová et al., 2018; Dixson, 2021; Valentova et al., 2021). Sex differences in body composition, notably muscularity and body size are some of the largest between women and men (Wells, 2007), which are often attributed to effects of sexual selection via mate choice (Dixson et al., 2010; Brooks et al., 2015). Lassek and Gaulin challenge this view using a large dataset from the NHANES and demonstrate small sex differences in stature, but marked differences in muscularity and body fat. Compared to women, men had 72% higher arm muscle, 65% greater muscle mass, and 36% greater lean muscle mass. Conversely, women store more than 1.6 times more body fat, the distribution of which is sexually dimorphic wherein women store greater gluteal femoral fat (i.e., the hips, buttock, and thighs) whereas men store more fat in the belly and midriff. Lassek and Gaulin conclude that women's body fat is naturally selected as reserves for neural development for the fetus during pregnancy, while men's muscularity and strength function for male-male competition for resources (Apicella, 2014), which may secondarily influence women's mate preferences for muscularity (Dixson et al., 2014; Lidborg et al., 2022).

One possible way to study sexual dimorphism is to view it developmentally. In a long-lived species like *Homo sapiens*, one would hypothesize that different life stages might be characterized by sexually differentiated adaptations in response to environmental challenges that impact the sexes differentially. Goetz et al. critique the field of Developmental Psychology, which, for many reasons, has minimized evolutionary and adaptationist explanations in favor of seeing socialization as explaining developmental changes. The authors argue that Developmental Psychology's failure to use ideas from Sexual Selection theory has led to an impoverished understanding of phenomena like children's playground behavior. There have, however, been some notable exceptions; Sexual Selection theory was utilized by Silverman and Eals (1992) to provide strong evidence for the hunter-gatherer theory (a truly integrative theory) of spatial skills development in children. They conclude by suggesting that an evolutionary and functional view of human mind and behavior once adopted by Developmental Psychology could take the field forward into a more accurate and robust understanding of developmental pathways.

Mezentseva et al. performed an experimental study, investigating gender differences in recognition of basic emotions (i.e., happiness, disgust, fear, and anger) among traditional Mongolian nomads of Southern Siberia, Tuvans. Unlike most previous studies, the authors reported no gender differences in the recognition of happiness, disgust, and fear in facial photographs of men and women. However, there were significant gender differences in anger recognition, where Tuvan men, compared to Tuvan women, were more accurate in labeling anger displays of men from their own population. The authors suggest a role for cultural conditioning in women's poor communication abilities. Even today, Mongolian cultural traditions encourage division of labor, where women's occupation and social environment are limited to the household, and prescribe limited contact between strangers of opposite sexes. Nevertheless, the reasons for Tuvan women's particular insensitivity to men's angry facial expressions remain poorly understood and require further research.

The results of an experimental study investigating trust and trustworthiness among strangers were presented by Rostovtseva et al.. The experiment combines a game-theoretic approach, video stimuli presentation, and the use of geometric morphometrics. In the paper, the authors have demonstrated that trust toward strangers is manifested at a remarkably high level, and is partly determined by the sex and appearance of an interaction partner. Generally, women elicited more trust in partners of both sexes, which supports previous findings. However, women more willingly trusted men with a more masculine facial appearance, while men themselves, on the contrary, did not trust them. According to the literature, men with highly masculinized faces tend to demonstrate less trustworthiness (Stirrat and Perrett, 2010). The authors suggest that one of possible explanations for the revealed female preferences could be that women find masculine male faces attractive for short-term sexual relationships, which may modulate their trusting behavior for the wrong reasons. The study illustrates the differential impact of male masculinization on the behavior of interaction partners of the same and the opposite sex. Despite a clear effect of sex and facial appearance on eliciting trust in strangers, actual trustworthiness was not associated either with sex or appearance. The revealed results raise the question of the adaptive value of the observed behavioral responses to partners' sex and sex-related facial traits.

## Papers on signaling, mate preferences, sexual strategies

Mailhos et al. investigated association between such male voice parameters as frequency (*F*_0_), formant dispersion, formant position or vocal tract length, and body size, as well as physical strength measured by handgrip strength. They confirmed some of the previous results on the relationship between vocal parameters and the key determinants of male physical formidability. They showed, for instance, that handgrip strength is negatively related to *F*_0_. It was also true when controlled for body height or weight. The authors also studied if voices of the weakest and the strongest men differed in attractiveness assessed by females, and did not find any association between male speakers' strength and perceived attractiveness by females. This study contributes to our understanding of the meaning of human male vocal characteristics as a potential cue or signal that could be important for the assessment of a men's physiological condition.

The conceptual paper, by Gangestad and Dinh, brings together questions of how and why women's sexual interests vary across the ovulatory cycle. One reliable empirical finding is that sexual desire increases when conception is more likely. But this is not the only time that women experience sexual desire, they are also interested in non-conceptive sex. The question is how to make sense of this finding as well as others in the literature. The authors suggest that we need to place more emphasis on the strategic analysis of behavior. It is likely that the circumstances that result in greater sexual interest during conceptive and non-conceptive phases of the ovulatory cycle are different. Sex in these different phases (i.e., mid-cycle vs. other phases) can have different functions and the different sexual interests may be motivated by different goals or strategies. Taking a more design feature or reverse-engineering approach is likely to be beneficial in combination with new theories to guide, not only future research on the role of extended sexuality in women, but our interpretation of the results.

Pisanski et al. investigated if mate preferences for body height in several countries (i.e., Canada, Cuba, Norway, and USA) depend on sexual strategies. They examined this via graphic stimuli containing metric indices. They confirmed the “male taller norm” in all countries. Relative to their own body height, men preferred shorter, and women taller sexual partners. What was, however, the most interesting result was that only men showed a different preference for their partner's height in relation to their sexual strategy. When choosing a partner for short-term relationships they preferred greater sexual dimorphism (i.e., relatively shorter partner than themselves) than when choosing a long-term partner. Women preferred the same partner height irrespective of the relationship context. This study adds new cross-cultural evidence for the importance of relationship context when mate preferences are concerned.

Sexual selection theory offers an essential framework for understanding the real world dynamics of gender roles, mate selection, sexual activity, and reproductive rates. Zhao et al. investigated these interactions in a sample of young Chinese adults, focusing on the relationship between androgyny and sexual activeness, with self-reported physical attractiveness, sexual motivation, and interpersonal relationships as mediators. Results were consistent with existing evidence suggesting that indeed there is a relationship between greater androgyny (i.e., lower adherence to gender roles as self-reported) and less self-reported heterosexual activity. The mediation results provide stimulating ideas for further research on these questions.

## Conclusion

This Research Topic offers new critical developments, fresh ideas, and solid results furthering Darwin's legacy on sexual selection applied to human evolution. Such accomplishment could only be possible with a large set of authors from diverse backgrounds and countries from North, Central and South America, Europe, Asia and Oceania. We present articles from a total of 56 authors from 17 countries: two from Austria, six from Brazil, seven from Canada, three from China, two from Cuba, two from the Czech Republic, one from France, two from Germany, one from Netherlands, one from New Zealand, one from Norway, three from Poland, six from Russia, four form Spain, one from the United Kingdom, three form Uruguay, and ten from the United States of America. In total, this rich combination of authors produced outputs from six different types of articles: seven original research articles, three perspective articles, two hypothesis and theory articles, four brief research reports, one conceptual analysis, and three reviews. We can safely conclude that this was the largest, most diverse, and highly promising sesquicentenary thematic issue of its kind.

Aligned with Darwin's *The Descent of Man, and Selection in Relation to Sex* original book (1871), the past celebratory pieces that followed, such as the classic centennial celebratory book titled *Sexual Selection and the Descent of Man, 1871–1971* edited by Campbell (1972), have also brought valuable insights and progress to the field. We hope we have helped assemble a strong set of empirical and theoretical articles in this Research Topic promising progress and future novel evolutionary hypotheses and conclusions.

## Author contributions

All authors listed have made a substantial, direct, and intellectual contribution to the work and approved it for publication.
